# *PERCC1*, a new member of the *Yap/TAZ*/*FAM181* transcriptional co-regulator family

**DOI:** 10.1093/bioadv/vbac008

**Published:** 2022-02-03

**Authors:** Luis Sanchez-Pulido, Siyang Jia, Carsten Gram Hansen, Chris P Ponting

**Affiliations:** 1 MRC Human Genetics Unit, Institute of Genetics and Cancer, University of Edinburgh, Edinburgh EH4 2XU, UK; 2 Centre for Inflammation Research, Institute for Regeneration and Repair, Queen's Medical Research Institute, Edinburgh bioQuarter, University of Edinburgh, Edinburgh EH16 4TJ, UK

## Abstract

**Motivation:**

Disrupted *PERCC1* gene expression causes an intractable congenital diarrhoea in infants. However, this gene’s molecular mechanism is unknown and no homologous proteins have been reported.

**Results:**

Our detailed evolutionary analysis of PERCC1 sequence reveals it to be a previously unappreciated member of the YAP/TAZ/FAM181 family of homologous transcriptional regulators. Like YAP and TAZ, PERCC1 likely interacts with DNA via binding to TEA/ATTS domain transcription factors (TEADs) using its conserved interface-2 and -3 sequences. We compare the expression patterns of *PERCC1* with those of *YAP, TAZ, TEADs*. Our report provides the identification and first in-depth bioinformatic analysis of a YAP/TAZ homologue, and a likely new regulator of the YAP/TAZ-TEAD transcriptional complex.

**Availability and implementation:**

The data underlying this article are available in UniProt Database.

**Supplementary information:**

Supplementary data are available at *Bioinformatics Advances* online.

## 1 Introduction

 YAP (Yes-associated protein) and TAZ (transcriptional coactivator with a PDZ-binding domain) are paralogues that both act as downstream effectors of the Hippo kinase cascade (Hansen *et al.*, 2015). They each lack DNA-binding domains but regulate transcriptional activity by shuttling to the nucleus where they bind TEAD (TEA/ATTS domain) transcription factors (Reggiani *et al.*, 2021), which are the main final nuclear effectors of the Hippo pathway (Vassilev *et al.*, 2001; Zhang *et al.*, 2009; Zhao *et al.*, 2008).

Structural and functional analysis have revealed detailed molecular insights into these YAP-TEAD and TAZ-TEAD heteromeric transcription complexes (Cao *et al.*, 2008; Lamar *et al.*, 2012; Li *et al.*, 2010; Mo *et al.*, 2015; Ota and Sasaki, 2008; Tian *et al.*, 2010; Vassilev *et al.*, 2001; Zhao *et al.*, 2008). Their TEAD-binding domain consists of three motifs (‘interface-1’, ‘interface-2’ and ‘interface-3’) that drape across the TEAD protein surface (Chen *et al.*, 2010; Li *et al.*, 2010; Mesrouze *et al.*, 2018; Pobbati *et al.*, 2012). Two additional paralogues, FAM181A and FAM181B, with interfaces-2 and -3 have also been described (Marks *et al.*, 2016) and structurally characterized (Bokhovchuk *et al.*, 2020).

*PERCC1* is a recently discovered gene, recognized only from its deletion in infants with intractable congenital diarrhoea who presented with nutrient malabsorption, multiple food intolerance and a failure to thrive (Oz-Levi *et al.*, 2019). It had previously been unannotated because of its expression being spatiotemporally restricted to rare cells present during stomach and intestine development (Oz-Levi *et al.*, 2019). *Percc1*–/– knockout mice recapitulate the human phenotype and this is rescued by expression of a *Percc1* transgene (Oz-Levi *et al.*, 2019). The protein's name refers to its proline (P) and glutamate (E)-rich sequence containing a putative coiled-coil (CC). Oz-Levi *et al.* (2019) reported PERCC1 orthologues across diverse vertebrate species but failed to identify homologous proteins with known function or structure. Consequently, the molecular mechanism of this enigmatic protein remains unknown. Here we show that PERCC1 is a YAP, TAZ and FAM181 homologue containing an intact TEAD-binding interfaces-2 and -3 (I2 and I3).

## 2 Results and discussion

### 2.1 Computational protein sequence analysis of the PERCC1 family

We started our analyses by conducting a JackHMMER iterative search with the human PERCC1 protein sequence of the Uniref50 protein sequence database (Eddy, 1996; Finn *et al.*, 2011; Wu *et al.*, 2006). As we iteratively accumulated PERCC1 orthologues we identified full-length homologous proteins from across the animal kingdom, not just in vertebrates (as before, Oz-Levi *et al.*, 2019) but also in earlier branching animals such as echinoderms, molluscs, annelids and chelicerates, and yet in neither nematodes (*Caenorhabditis elegans*) nor hexapods (*Drosophila melanogaster*) (Supplementary Fig. S1).

As input for our domain-hunting strategy, we first constructed a full-length multiple sequence alignment of these PERCC1 family members (Supplementary Fig. S1). We next used regions of this alignment that exhibited the highest levels of conservation as queries for profile-to-sequence (HMMer) and profile-to-profile comparisons (HHpred) (Supplementary Fig. S1) (Eddy, 1996; Finn *et al.*, 2011; Söding *et al.*, 2005).

**Fig. 1.**
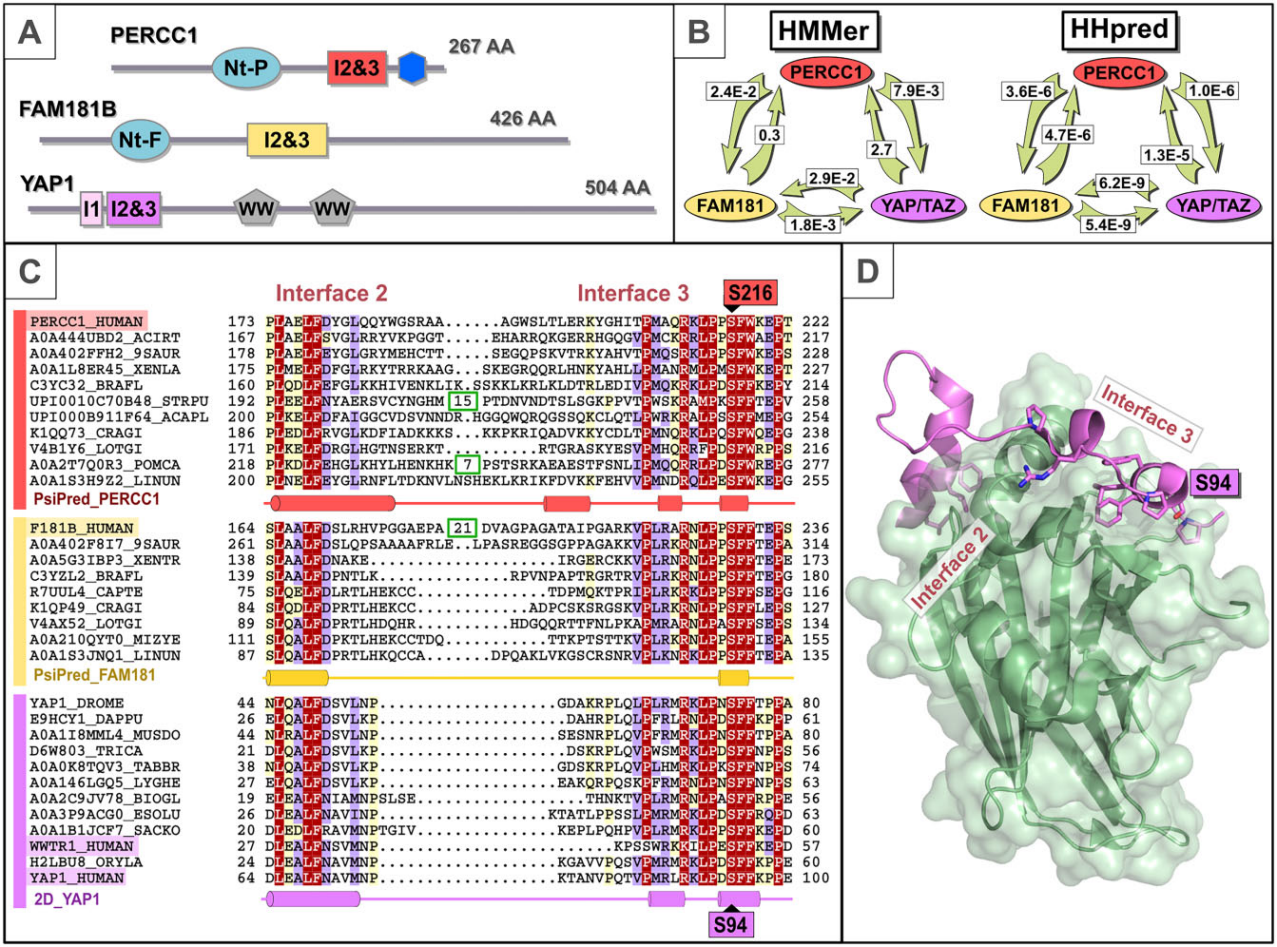
Computational protein sequence analysis of PERCC1 family. (**A**) Schematic representation of the domain architectures of PERCC1*,* FAM181B and YAP1. Consecutive interfaces 2 and 3 motifs (I2&3) in human PERCC1*,* FAM181B and YAP1 proteins are represented by coloured rectangles in red, yellow and purple, respectively. Conserved N-terminal regions in PERCC1 (Nt-P) and FAM181 (Nt-F) families are represented as cyan ovals (see Supplementary Material, Supplementary Figs S1 and S5). A conserved C-terminal region in PERCC1 is represented as a blue hexagon. YAP family has an additional small motif preserved in its interaction with TEAD, called interface 1 (I1) here represented by a pink rectangle. WW domains in YAP1 are indicated by grey pentagons (Chen *et al.*, 2010; Li *et al.*, 2010; Mesrouze *et al.*, 2018; Pobbati *et al.*, 2012). (**B**) Sequence conservation analysis of interface 2 and 3 (I2&3) motifs among PERCC1*,* FAM181B and YAP1 families. Left: HMMer profile-versus-sequence comparison *E*-values (shown in white boxes) from profile search results (Eddy, 1996; Finn *et al.*, 2011). Right: HHpred profile-versus-profile comparison *E*-values from global profile search results (Söding *et al.*, 2005). In both, arrows indicate the profile search direction. (**C**) Representative multiple sequence alignment of two consecutive TEAD interaction motifs (‘interfaces 2 and 3’) in PERCC1, FAM181B and YAP/TAZ families. Protein families are indicated by coloured bars at the left of the alignment: PERCC1, FAM181B and YAP/TAZ are indicated in red, yellow and purple, respectively. Limits of protein sequences included in the alignment are indicated by flanking residue positions. Numbers inside green boxes represent excised unconserved sequence. Secondary structure predictions acquired using PsiPred (Jones, 1999) were performed independently for PERCC1 and FAM181 families (shown as PsiPred_Percc1 and PsiPred_Fam181 lanes, respectively); these predictions are consistent with the known secondary structure of the human YAP1 protein shown in the 2D_YAP1 lane. Cylinders indicate α and 310 helices. The alignment was generated with T-Coffee (Notredame *et al.*, 2000) and presented with the program Belvu using a colouring scheme indicating the average BLOSUM62 scores (which are correlated with amino acid conservation) of each alignment column: red (>3), violet (between 3 and 0.8) and light yellow (between 0.8 and 0.2) (Sonnhammer and Hollich, 2005). Human YAP1 serine 94, a known target of AMPK mediated phosphorylation, and its corresponding position in human PERCC1 (serine 216), are labelled in purple and red, respectively. Sequences are named according to their UniProt identification (Wu *et al.*, 2006). Species abbreviations: 9SAUR, *Paroedura picta*; ACAPL, *Acanthaster planci*; ACIRT, *Acipenser ruthenus*; BIOGL, *Biomphalaria glabrata*; BRAFL, *Branchiostoma floridae*; CAPTE, *Capitella teleta*; CRAGI, *Crassostrea gigas*; DAPPU, *Daphnia pulex*; DROME, *Drosophila melanogaster;* ESOLU, *Esox lucius*; HUMAN, *Homo sapiens*; LINUN, *Lingula unguis*; LOTGI, *Lottia gigantea*; LYGHE, *Lygus hesperus*; MIZYE, *Mizuhopecten yessoensis*; MUSDO, *Musca domestica*; ORYLA, *Oryzias latipes*; POMCA, *Pomacea canaliculata*; SACKO, *Saccoglossus kowalevskii*; STRPU, *Strongylocentrotus purpuratus*; TABBR, *Tabanus bromius*; TRICA, *Tribolium castaneum*; XENLA, *Xenopus laevis*; XENTR, *Xenopus tropicalis*. (**D**) Location of interfaces 2 and 3 in the YAP1 peptide and TEAD heterodimer structure. Cartoon representation of human YAP1 peptide and TEAD secondary structures is shown coloured in purple and green, respectively (PDB: 3KYS) (Li *et al.*, 2010). Regions corresponding to interfaces 2 and 3 are labelled. Only highly conserved residues among PERCC1, FAM181B and YAP/TAZ (red columns in Fig. 1C alignment) are shown as sticks (LxxLF in Interface 2 and PxxxRxLPxSFFxxP in Interface 3). Human YAP1 serine 94 is labelled. Structure was rendered using Pymol (http://www.pymol.org)

Unexpectedly, these database searches yielded statistically significant sequence similarity between PERCC1 and the YAP/TAZ/FAM181 family of transcriptional co-regulators. For example, a profile-to-sequence search against the Uniref50 database with the central conserved region of the PERCC1 family (corresponding to amino acids 173–222 of human PERCC1) as query, identified first a known YAP/TAZ family member from the chelicerate *Sarcoptes scabiei* (UniProt: A0A132A5J4_SARSC) with *E *=* *7.9×10^−3^, and then a known FAM181 family member from the annelid *Capitella teleta* (UniProt: R7UUL4_CAPTE) with *E *=* *0.024 (Fig. 1B).*E* is the number of alignments of similar score, or better, that are expected to be found in the database search by chance. Consequently, these low HMMer *E-*values (*E *<* *0.1) are indicative of significant sequence similarity between PERCC1 and YAP/TAZ/FAM181, and thereby of their homology, the vertical descent of sequence from a common ancestral sequence.

Next, we used HHpred (Söding *et al.*, 2005) to undertake a global profile-to-profile search against the PDB70 database. A search with the central conserved region of PERCC1 yield significant similarity to human YAP (PDB: 6GEI_L) (Mesrouze *et al.*, 2018) with *E *=* *1.0×10^−6^ and a true-positive probability of 98%. We then tested for self-consistent and reciprocal sequence relationships between conserved regions of each of the YAP/TAZ, FAM181 and PERCC1 families, again using HHpred (Söding *et al.*, 2005). Once more, statistically significant and reciprocal sequence similarities were observed between these regions (in all cases E < 5.0×10^−4^; Fig. 1B). These strong statistical relationships further substantiate the homology between PERCC1 and both YAP/TAZ and FAM181 families. The region identified as conserved between PERCC1 and YAP/TAZ/FAM181 families corresponds precisely with the interfaces-2 and -3 TEAD-interacting region of YAP (Fig. 1C and D) (Chen *et al.*, 2010; Li *et al.*, 2010; Mesrouze *et al.*, 2018; Pobbati *et al.*, 2012).

Additional sequence similarity searches allowed us to confirm the relationship previously described by Mesrouze *et al.* (2020). Here, the authors identified structural and sequence similarity between YAP and Vestigial families' TEAD-interacting motifs (Supplementary Fig. S2).

The small size of PERCC1’s evolutionarily conserved regions and its low amino acid composition complexity might explain why these remote relationships had previously gone unnoticed.

Next, we generated a Swiss-Model PERCC1/TEAD heterodimer homology model (Waterhouse *et al.*, 2018), using as template the crystal structure of human YAP and TEAD complex (PDB: 3KYS) (Li *et al.*, 2010). Predicted association of PERCC1 to TEAD1 was supported by the negative value of solvation-free energy gain upon interface interaction, with values comparable to those obtained in the analysis of known structure complexes in the family (Supplementary Fig. S3) (Kaan *et al.*, 2017; Krissinel and Henrick, 2007; Li *et al.*, 2010; Xue *et al.*, 2016). Gibbs’ free energy of binding predictions using PISA (Proteins, Interfaces, Structures and Assemblies) tool for YAP, TAZ and PERCC1 in interaction with TEAD were -17.5, -18.5 and -20.3 kcal/mol, respectively (Krissinel and Henrick, 2007).

It is notable that human YAP serine 94, a known target of AMPK (AMP-activated protein kinase) mediated phosphorylation (Mo *et al.*, 2015), is also fully conserved in PERCC1 (serine 216 in human PERCC1) (Fig. 1C). This serine is required for YAP’s binding to TEAD (Li *et al.*, 2010) and its phosphorylation disrupts this interaction (Mo *et al.*, 2015). In PERCC1, this serine residue is completely conserved across 239 diverse placental mammals (Zoonomia Consortium, 2020) as well as in more divergent animal species (Fig. 1C). This implies that its participation in protein-binding, functional importance and/or phosphorylation provides a regulatory mechanism not just in YAP but also in PERCC1.

*PERCC1* gene expression is low and narrow in tissue- and cell-type range, which likely explains why it escaped detection as a protein-coding gene until recently. In none of 27 human reference tissues did its mRNA expression exceed 1 RPKM (Reads Per Kilobase of transcript per Million mapped reads) (Fagerberg *et al.*, 2014). It achieves modest expression in gastric G cells and duodenal enteroendocrine cells (Oz-Levi *et al.*, 2019), in particular in duodenal crypts (Stine *et al.*, 2019). *Percc1* is also robustly expressed in Stmn1+ isthmal stem cells and Pgc+ gastric cells in which *Tead1, Tead2, Tead3* and *AMPK* subunits, as well as *Yap1* and *Taz*, are also expressed (Han *et al.*, 2019; Papatheodorou *et al.*, 2020) (Supplementary Fig. S4).

Its YAP/TAZ-like TEAD-interacting region (i.e. interfaces-2 and -3), and its mRNA’s co-expression with *Tead1/2/3* in cells of the gastric corpus, indicate that PERCC1 protein might act as a YAP/TAZ-like transcriptional coactivator bound to TEAD proteins in these cells. Such interactions are consistent with previous described roles of TEAD transcription factors in intestinal cell development and regeneration (Guillermin *et al.*, 2021; Kriz and Korinek, 2018; Wang *et al.*, 2016) and the observed PERCC1 gastrointestinal phenotype (Oz-Levi *et al.*, 2019). *PERCC1*-expressing cell lines that adequately model gastrointestinal stem cells are required to address these mechanistic hypotheses and are not yet available.

### 2.2 Computational protein sequence analysis of the FAM181 family

Following our PERCC1 analysis, we performed a similar protocol of domain and motif identification for the FAM181 protein family and identified two evolutionarily conserved regions, one at their N-termini and the other corresponding to the previously described TEAD interacting-region (Bokhovchuk *et al.*, 2020; Marks *et al.*, 2016) (Supplementary Fig. S5).

The FAM181 N-terminal conserved region is homologous to a region of an experimentally uncharacterized human protein, C19orf85, which is thus a previously unrecognized FAM181 family member (Supplementary Fig. S5). For example, a profile-to-sequence search against Uniref50 database with the FAM181 N-terminal conserved (corresponding to amino acids 59–106 of human FAM181B) as query, identified tortoise C18orf85 (UniProt: A0A452HNJ8_9SAUR) with *E = *1.8×10^−8^. The reciprocal sequence similarity search yielded concordant results: a profile-to-sequence search against Uniref50 database with the C19orf85 N-terminal conserved region (corresponding to amino acids 12–61 of human C19orf85) as query, identified a FAM181 homolog from the fish *Oreochromis niloticus* (UniProt: I3L013_ORENI) with an *E* = 2.0×10^−6^ (Eddy, 1996; Finn *et al.*, 2011). C19orf85 is unlike other FAM181 family members in lacking recognizable TEAD interaction motifs (Supplementary Fig. S5). Human *C19ORF85* also shows a narrow expression pattern, mainly in the gastrointestinal tract, in particular the colon (GTEx v8) (GTEx Consortium, 2020).

Finally, the N-terminal conserved regions of FAM181, PERCC1 and C19orf85 were discovered as being homologous within a predicted α helix (Supplementary Fig. S6). PERCC1 thus shares a similar domain architecture with the FAM181 protein family (Fig. 1) which implies similarities in their function.

AlphaFold, a recently developed machine learning approach, usually predicts protein structures from sequence with high accuracy (Jumper *et al.*, 2021; Tunyasuvunakool *et al.*, 2021). Nevertheless, AlphaFold structural predictions of TEAD-interacting motifs I2–I3 sequence in PERCC1 and FAM181B do not superimpose on these motifs in the known YAP structure (Supplementary Fig. S7 and Supplementary Video). This is likely due to the great flexibility and variable length of the linker that connects I2 and I3 in these proteins (Fig. 1C). Our analysis is thus an unusual example where Alphafold is outperformed by conventional protein sequence conservation analysis.

## 3 Conclusion

Our analysis reveals human PERCC1 to be a new member of the YAP/TAZ/FAM181 family of transcriptional regulator homologues. Amino acid conservation patterns indicate that PERCC1 is a previously unappreciated TEAD-interacting protein whose binding might be regulated by AMPK-mediated phosphorylation at serine 216. This computational prediction of PERCC1 function helps to formulate hypotheses regarding the molecular mechanisms by which it regulates vertebrate gastrointestinal system development.

## Supplementary Material

vbac008_Supplementary_Data
