# Supplementary material for: PERCC1, a new member of the Yap/TAZ/FAM181 transcriptional co-regulator family
Source: Bioinform Adv. 2022 Feb 3;2(1):vbac008. doi: 10.1093/bioadv/vbac008 (PMC9710580; doi:10.1093/bioadv/vbac008)
Supplement: vbac008_Supplementary_Data [file vbac008_supplementary_data.zip › SupplementalMaterialPercc1.pdf]

Supplementary Material

Computational protein sequence and structural analysis methods.

Multiple sequence alignments were generated with the program T-Coffee using default parameters (Notredame *et al.,* 2000), slightly refined manually and visualized with the Belvu program (Sonnhammer and Hollich, 2005). Profiles of the alignment as hidden Markov models (HMMs) were generated using HMMer (Eddy, 1996; Finn *et al.,* 2011). Profile-based sequence searches were performed against the Uniref50 protein sequence database (Wu *et al.,* 2006) using HMMsearch (Eddy, 1996; Finn *et al.,* 2011). Profile-to-profile comparisons were performed using HHpred profile-to-profile comparisons (Söding *et al.,* 2005). Profile-to-sequence (HMMer) and Profile-to-profile (HHpred) matches were evaluated in terms of an *E*-value, which is the expected number of non-homologous proteins with a score higher than that obtained for the database match. An *E*-value much lower than one indicates statistical significance. Sequence similarity searches for with proteins of known structure were performed with HHpred against PDB70. The PDB70 database contains profile hidden Markov models (HMMs) for representative sequences, clustered to 70% maximum pairwise sequence identity to reduce redundancy, drawn from the PDB database (Söding *et al.,* 2005). Secondary structure predictions were performed using PsiPred (Jones, 1999). PISA and PRODIGY tools were used to calculate chemical and structural properties of protein-protein interfaces (Krissinel and Henrick, 2007; Xue *et al.*, 2016). Swiss-Model web server was used for heterodimer homology modelling (Waterhouse *et al.*, 2018). Figures were generated using Inkscape (http://inkscape.org/). Structures were analysed using Pymol (http://www.pymol.org).

Figure S1. Evolutionary conserved regions in PERCC1 family.

Top: Schematic representation of the PERCC1 domain architecture. A conserved N-terminal region in PERCC1 (Nt-P) is represented as a cyan oval. Consecutive interfaces 2 and 3 motifs (I2&I3) in human PERCC1 is represented by a red rectangle. A conserved C-terminal region in PERCC1 is represented as a blue hexagon.

Bottom: Representative multiple sequence alignment of the PERCC1 family generated with the program T-Coffee using default parameters (Notredame *et al.,* 2000). The amino acid colouring scheme indicates the average BLOSUM62 score (correlated to amino acid conservation) in each alignment column: black (greater than 3), grey (between 3 and 1.5) and light grey (between 1.5 and 0.5). Conserved regions in the PERCC1 protein family are highlighted in different colours: N-terminal conserved box (Nt-P), interfaces 2 and 3 motifs (I2&3), and a C-terminal conserved region are coloured in cyan, red, and blue, respectively. Sequences are named according to their UniProt identifier (Wu *et al.,* 2006). Species abbreviations: 9SAUR, *Paroedura picta*; ACAPL, *Acanthaster*

planci; ACIRT, *Acipenser ruthenus*; BRAFL, *Branchiostoma floridae*; CRAGI, *Crassostrea gigas*; DANRE, *Danio rerio*; HUMAN, *Homo sapiens*; LINUN, *Lingula unguis*; LOTGI, *Lottia gigantea*; MOUSE, *Mus musculus*; POMCA, *Pomacea canaliculata*; STRPU, *Strongylocentrotus purpuratus*; XENLA, *Xenopus laevis*.

Figure S2. Evolutionarily conserved regions in Vestigial family proteins.

A: Cartoon representations of Alphafold 3D model of human VGLL2 protein (Tunyasuvunakool *et al.*, 2021). Coloured in magenta, yellow, and red are the interface motifs I1, I2, and I3, respectively. Inset top-right: structural superposition of Alphafold models for I3 motifs in VGLL2 (red) and VGLL3 (pink), and the known crystallographic structure of the I3 motif in Drosophila Vg protein (blue) (PDB: 6Y20) (Mesrouze *et al.*, 2020). VGLL2 serine 144 (equivalent to human YAP1 serine 94) is labelled.

B: Schematic representation of the domain architectures for four Vestigial human paralogs: VGLL1, VGLL2, VGLL3, and VGLL4. Consecutive interfaces 1, 2, and 3 motifs in Vestigial paralogs are represented by magenta, yellow, and red rectangles, respectively. As for the FAM181 protein family (FAM181A, FAM181B and C19ORF85), evolution of the Vestigial family has involved the loss and/or degeneration of subsets of these interface motifs among paralogues, resulting in divergent modes of TEAD regulation (Figure S5).

C: Representative multiple sequence alignments of Vestigial conserved regions adapted from Mesrouze *et al.* (Mesrouze *et al.*, 2020). The amino acid colouring scheme indicates the average BLOSUM62 score (correlated to amino acid conservation) in each alignment column: black (greater than 3), grey (between 3 and 1.5) and light grey (between 1.5 and 0.5). Conserved regions corresponding to I1, I2, and I3 motifs are highlighted in magenta, yellow, and red, respectively. Sequences are named according to their UniProt identifier (Wu *et al.*, 2006). Species abbreviations: DROME, *Drosophila melanogaster*; HUMAN, *Homo sapiens*; LINUN, *Lingula unguis*; MOUSE, *Mus musculus*; TENMO, *Tenebrio molitor*; XENLA, *Xenopus laevis*.

Figure S3. PERCC1/TEAD heterodimer homology model and interface analysis.

Left: Cartoon representation of human PERCC1 and TEAD structural model is shown coloured in red and green, respectively (Waterhouse *et al.*, 2018). Regions corresponding to interfaces 2 and 3 are labelled. All PERCC1 residues are shown in sticks. Structure was rendered using Pymol (http://www.pymol.org).

Right: For the analysis of interfaces-2 and -3 in human PERCC1/TEAD hypothetical complex we used the heterodimer homology model generated by Swiss-Model (shown on the left) (Waterhouse

et al., 2018). For the analysis of interfaces-2 and -3 in human YAP/TEAD and mouse TAZ/TEAD complexes we used the protein data bank crystal structures of 3KYS (chains A and B) and 5GN0 (chains B and G), respectively (Li *et al.,* 2010; Kaan *et al.,* 2017). PISA was used to calculate Gibbs free energy of binding and interface area of these protein-protein interfaces (Krissinel and Henrick, 2007). PRODIGY was used to calculate their dissociation constants (Xue *et al.*, 2016).

Figure S4. t-SNE plots from Percc1 and putative functionally related genes.
t-SNE plots were download from the Single Cell Expression Atlas EBI Server (release 18) (Papatheodorou *et al.*, 2020) and are based on the single cell RNA-seq experiment E-MTAB-6879 (Han *et al.*, 2019). E-MTAB-6879 experiment is a single cell RNA-seq of Stmn1+ isthmal stem cells and Pgc+ gastric chief cells in the mouse gastric corpus epithelium (Han *et al.*, 2019). Top left: annotated cell clusters. Subsequent plots: single cell expression levels for selected genes (Ensembl IDs shown in parentheses): Percc1 (ENSMUSG00000114245), Yap1 (ENSMUSG00000053110), Taz (ENSMUSG00000027803), Tead1 (ENSMUSG00000055320), Tead2 (ENSMUSG00000030796), Tead3 (ENSMUSG00000002249), and Tead4 (ENSMUSG00000030353). AMPK is a serine/threonine heterotrimeric kinase composed of an alpha catalytic subunit (Prkaa1 or Prkaa2) bound to a beta (Prkab1 or Prkab2) and gamma (Prkag1, Prkag2 or Prkag3) regulatory subunits: Prkaa1 (ENSMUSG00000050697), Prkaa2 (ENSMUSG00000028518), Prkab1 (ENSMUSG00000029513), Prkab2 (ENSMUSG00000038205), Prkag1 (ENSMUSG00000067713), Prkag2 (ENSMUSG00000028944), and Prkag3 (ENSMUSG00000006542).

Figure S5. Evolutionary conserved regions in FAM181 family.
Top: Schematic representation of the domain architecture for three FAM181 human paralogs: FAM181B, FAM181A, and C19orf85. A conserved N-terminal region (Nt-F) in FAM181 family is represented as a cyan oval. Consecutive interfaces 2 and 3 motifs (I2&3) in human FAM181B is represented by a yellow rectangle. The I3 motif identified in FAM181A is represented by a yellow rectangle (Bokhovchuk *et al.,* 2020; Marks *et al.,* 2016). The conserved C-terminal region in human C19orf85 protein is represented as an unlabelled orange circle.
Bottom: Representative multiple sequence alignments of FAM181 conserved regions generated with the program T-Coffee using default parameters (Notredame *et al.,* 2000). The amino acid colouring scheme indicates the average BLOSUM62 score (correlated to amino acid conservation) in each alignment column: black (greater than 3), grey (between 3 and 1.5) and light grey (between 1.5 and 0.5). Conserved regions corresponding to I2 and I3 motifs are highlighted in yellow. We were unable to find sufficient statistical significance of sequence similarity to justify homology for

the interface 2 motif (I2) within the FAM181A subfamily. Sequences are named according to their UniProt identifier (Wu *et al.,* 2006). Species abbreviations: 9SAUR, *Paroedura picta*; BRAFL, *Branchiostoma floridae;* CAPTE, *Capitella teleta*; CHAVO, *Charadrius vociferus;* CRAGI, *Crassostrea gigas*; FUKDA, *Fukomys damarensis*; HUMAN, *Homo sapiens*; LINUN, *Lingula unguis*; LOTGI, *Lottia gigantea*; MIZYE, *Mizuhopecten yessoensis*; MOUSE, *Mus musculus*; NANPA, *Nanorana parkeri*; OPHHA, *Ophiophagus hannah*; PELSI, *Pelodiscus sinensis*; RABIT, *Oryctolagus cuniculus*; VOMUR, *Vombatus ursinus*; XENLA, *Xenopus laevis;* XENTR, *Xenopus tropicalis.*

Figure S6. Evolutionarily conserved N-terminal region in PERCC1 and FAM181 families. Representative multiple sequence alignment generated with the program T-Coffee using default parameters (Notredame *et al.,* 2000). A HHpred profile-to-profile search against Pfam profile database using as input the first part of the PERCC1 N-terminal conserved region (corresponding to human PERCC1 from 79 to 106) found FAM181 Pfam database entry (Family: FAM181/PF15238) with a true-positive probability of 73.2% and $E = 0.28$ (Söding *et al.,* 2005). The alignment was presented with the program Belvu using a colouring scheme indicating the average BLOSUM62 scores (which are correlated with amino acid conservation) of each alignment column: red (>1.7), violet (between 1.7 and 1) and light yellow (between 1 and 0.3) (Sonnhammer and Hollich, 2005). Protein families are indicated by coloured bars to the left of the alignment: PERCC1 and FAM181B are indicated in red, and yellow, respectively. The limits of the protein sequences included in the alignment are indicated by flanking residue positions. Secondary structure predictions using PsiPred (Jones, 1999) were performed independently for PERCC1 and FAM181 families, shown in PsiPred_Percc1 and PsiPred_Fam181 lanes, respectively. Cylinders indicate α helices. Sequences are named according to their UniProt identification (Wu *et al.,* 2006). Species abbreviations can be found in the legends to Figure S1 and S5.

Figure S7. Structural superposition of I2-I3 motifs in PERCC1, FAM181B, and YAP. Structural superposition of Alphafold2 models for motif I2-I3 in PERCC1 (red), FAM181B (yellow), and YAP (purple) (Tunyasuvunakool *et al.,* 2021). Structural similarity is limited to the I3 motif (also known as the omega loop). Regions correspond to those in the alignment shown in Figure 1C: positions 173 to 222 in PERCC1, 164 to 236 in Fam181B, and 64 to 100 in YAP1. Shown as sticks are two conserved consecutive residues in I2 (Leu-Phe) corresponding to residues 177-178, 168-169, and 68-69 in PERCC1, FAM181B, and YAP, respectively. Also shown in sticks are the I3 conserved serines 216, 230, and 94 in PERCC1, FAM181B, and YAP, respectively.

Supplemental Video. Alphafold 3D models.

The video includes cartoon representations for four Alphafold 3D models of human proteins: PERCC1 (top-left), FAM181B (top-right), YAP1 (down-left), and C19ORF85 (down-right (Tunyasuvunakool *et al.,* 2021). Coloured in red are the interface motifs I2 and I3 (shown in Figure 1C). Coloured in green is the N-terminal evolutionarily conserved region in PERCC1 (Figure S1, region Nt-P), and in cyan the N-terminal evolutionarily conserved region in FAM181 (Figure S5, region Nt-F). Coloured in blue is the C-terminal evolutionarily conserved region in PERCC1 (blue hexagon in Figure S1). Coloured in orange is the C-terminal evolutionarily conserved region in the C19ORF85 subfamily (orange oval in Figure S5). Lastly, coloured in black are the two WW domains found in YAP1 (grey pentagons in Figure 1A). Alphafold models were downloaded from UniProt and visualised using Pymol (http://www.pymol.org).
