# Supplementary figures and images for: PERCC1, a new member of the Yap/TAZ/FAM181 transcriptional co-regulator family
Source: Bioinform Adv. 2022 Feb 3;2(1):vbac008. doi: 10.1093/bioadv/vbac008 (PMC9710580; doi:10.1093/bioadv/vbac008)

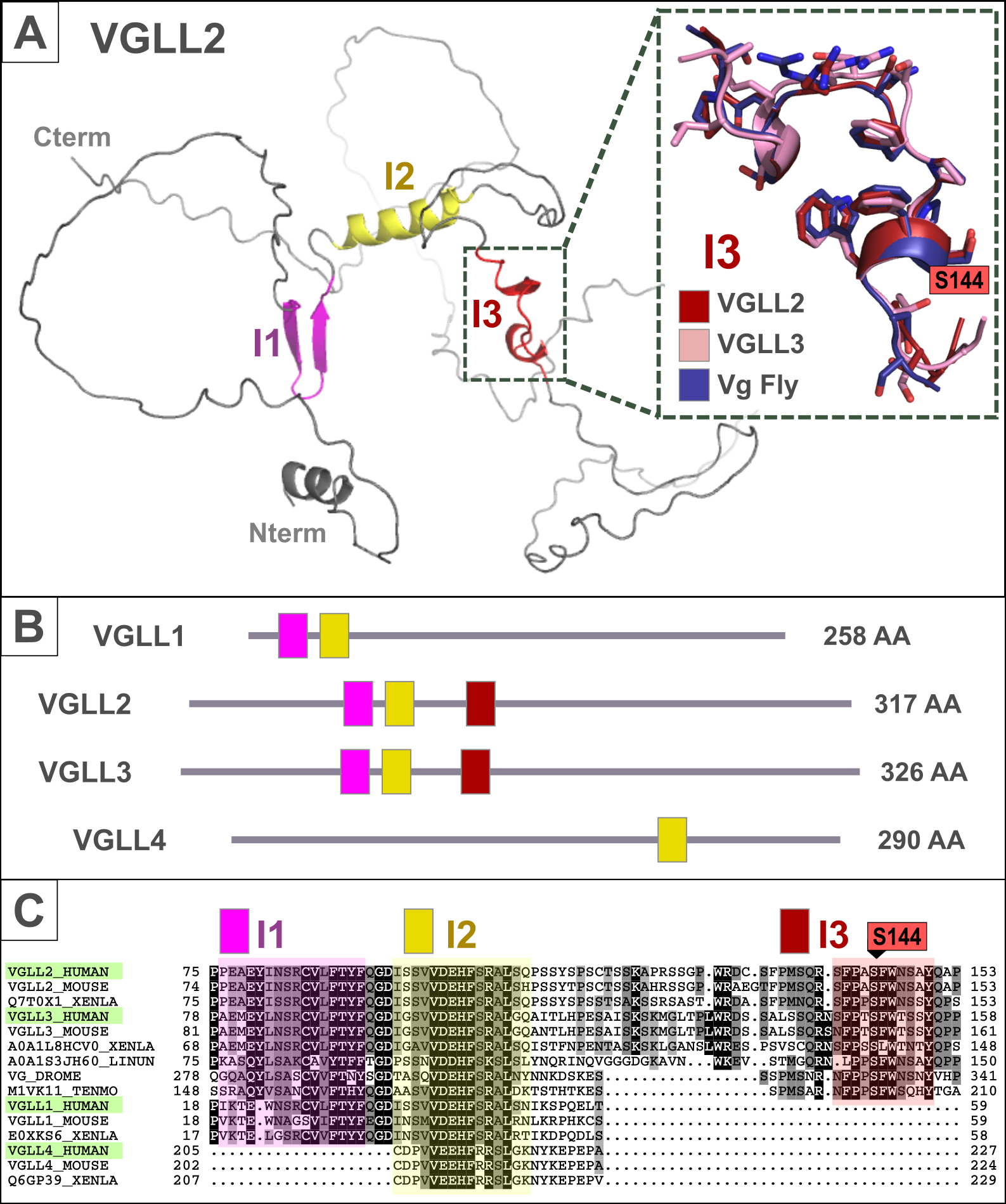

Supplement: vbac008_Supplementary_Data [file vbac008_supplementary_data.zip › FigureS2Percc1.png]

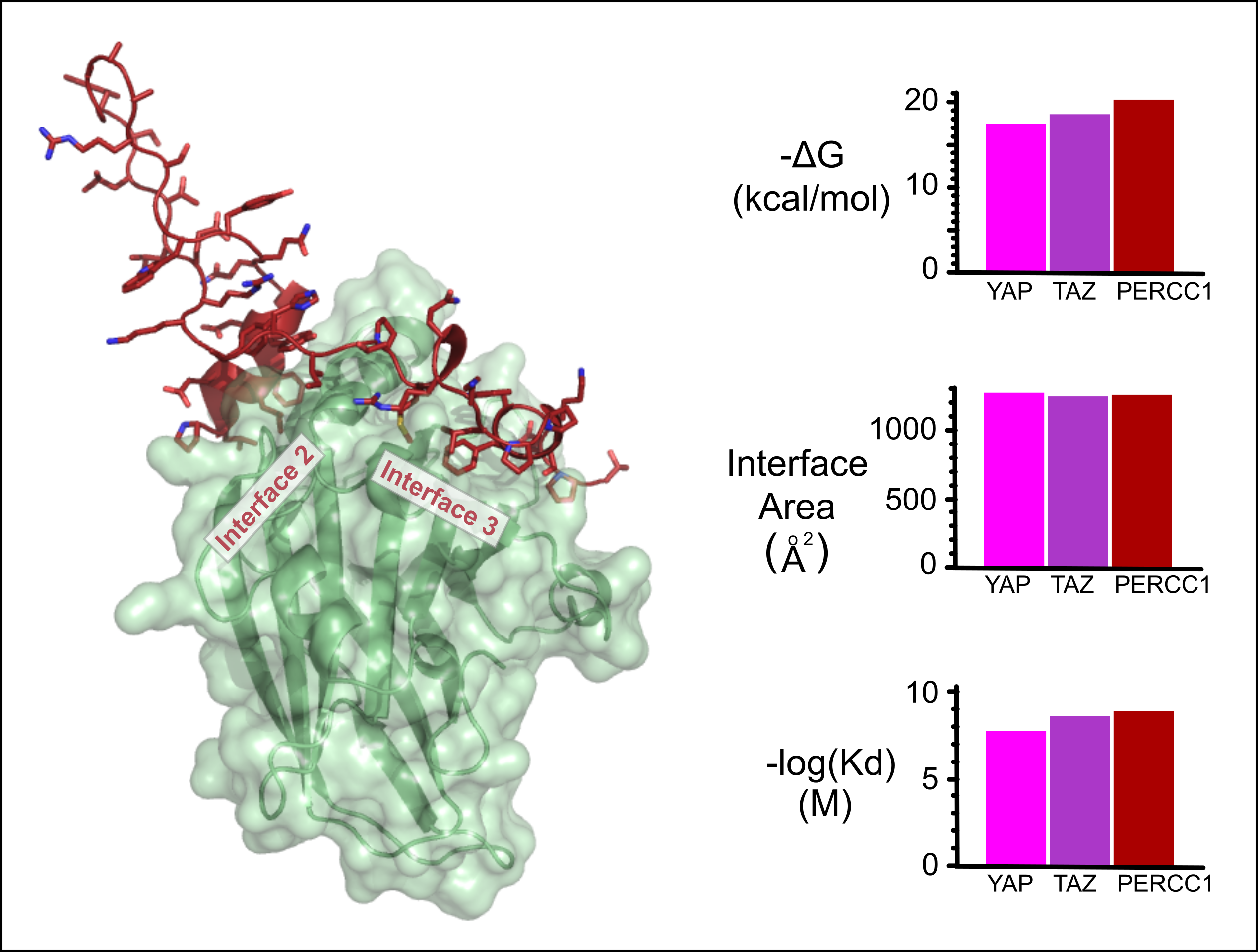

Supplement: vbac008_Supplementary_Data [file vbac008_supplementary_data.zip › FigureS3Percc1.png]

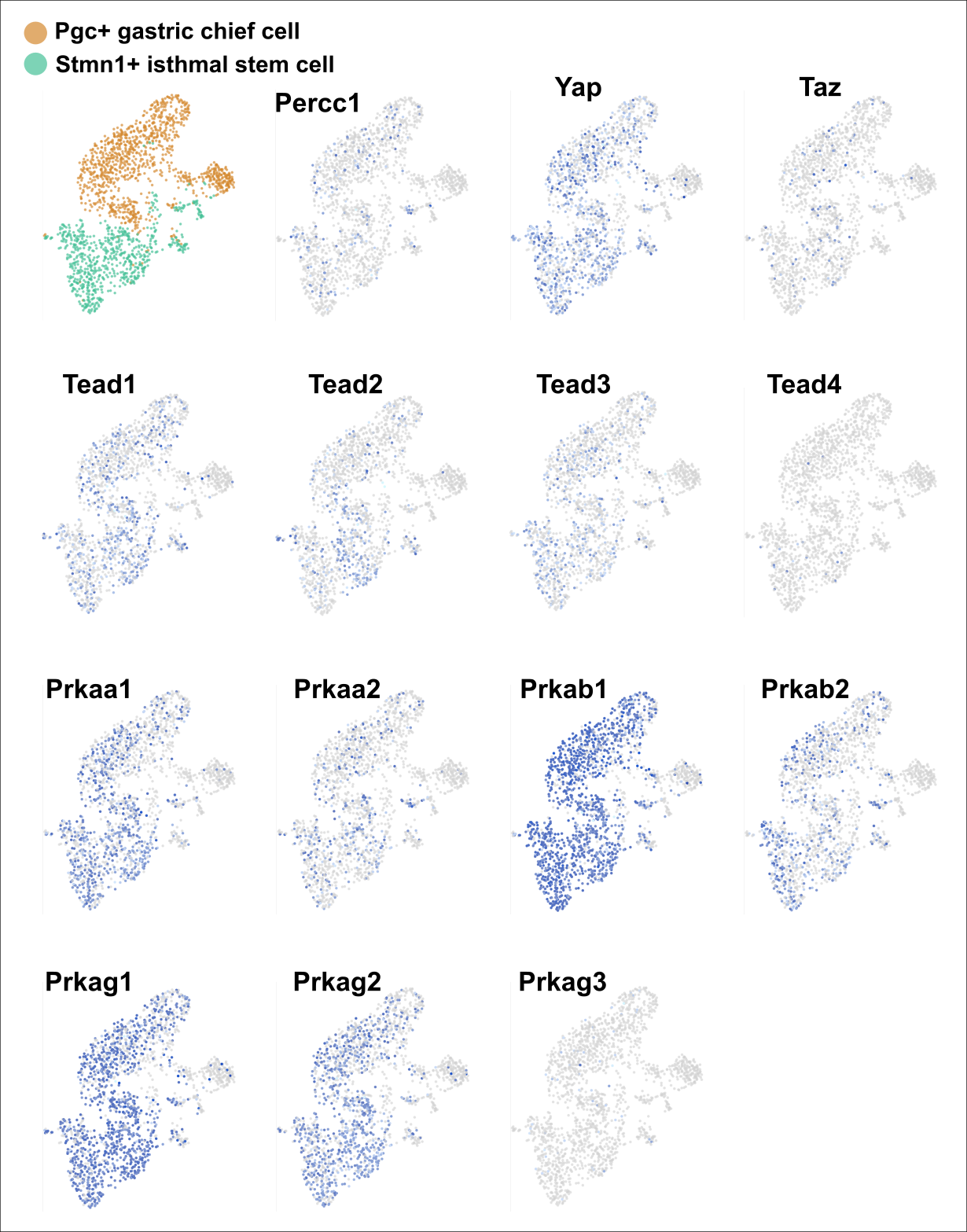

Supplement: vbac008_Supplementary_Data [file vbac008_supplementary_data.zip › FigureS4Percc1.png]

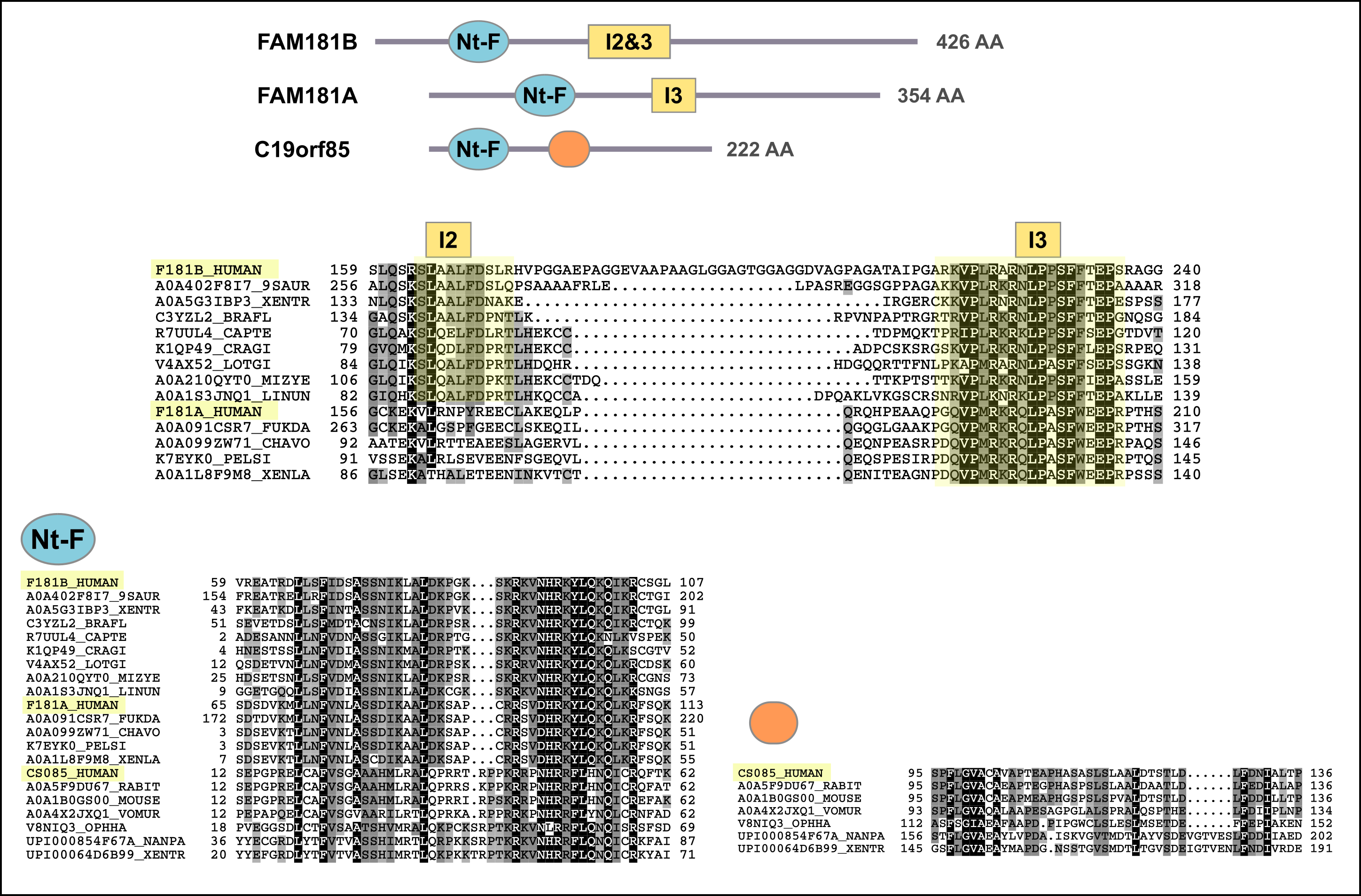

Supplement: vbac008_Supplementary_Data [file vbac008_supplementary_data.zip › FigureS5Percc1.png]

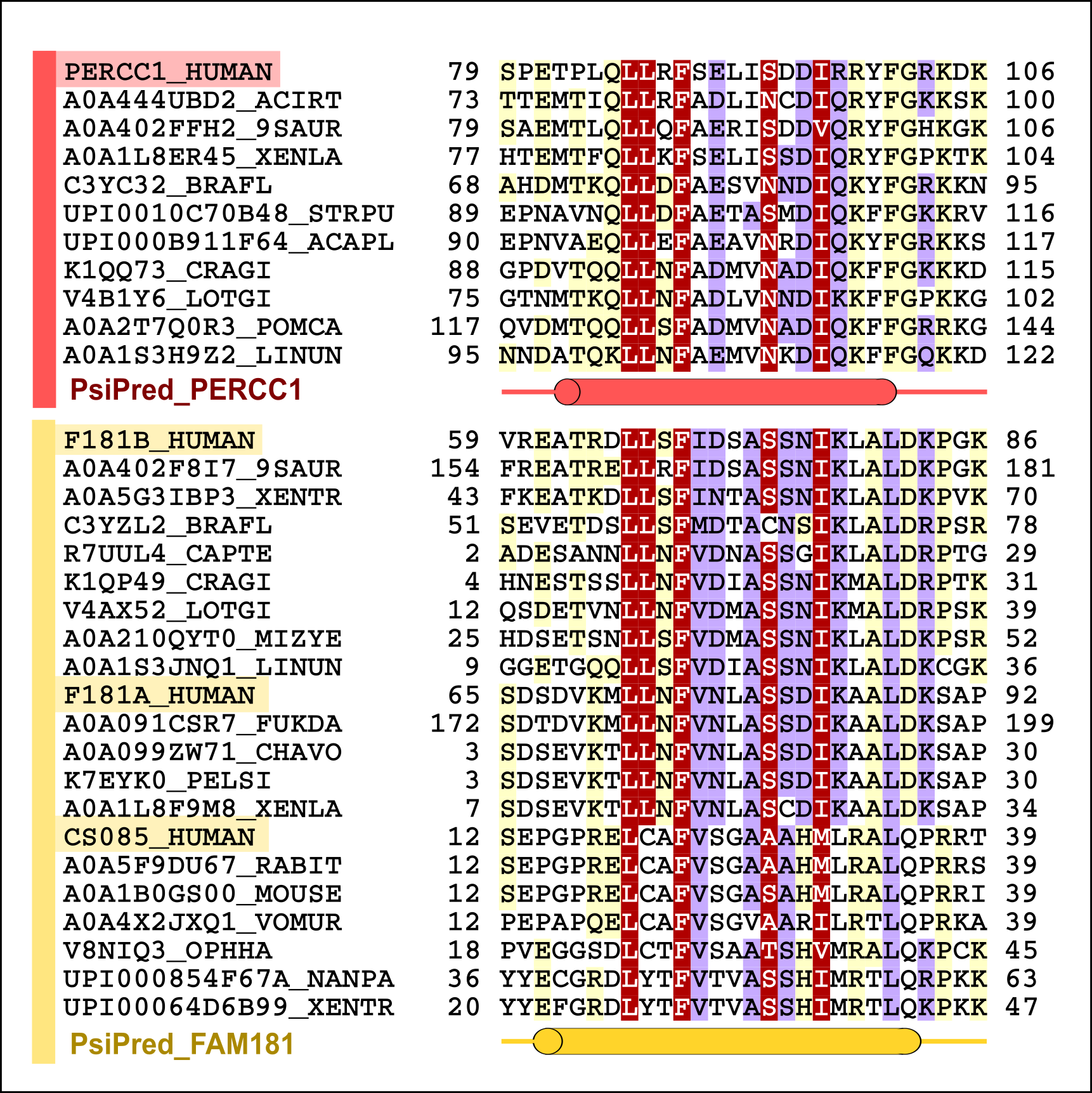

Supplement: vbac008_Supplementary_Data [file vbac008_supplementary_data.zip › FigureS6Percc1.png]

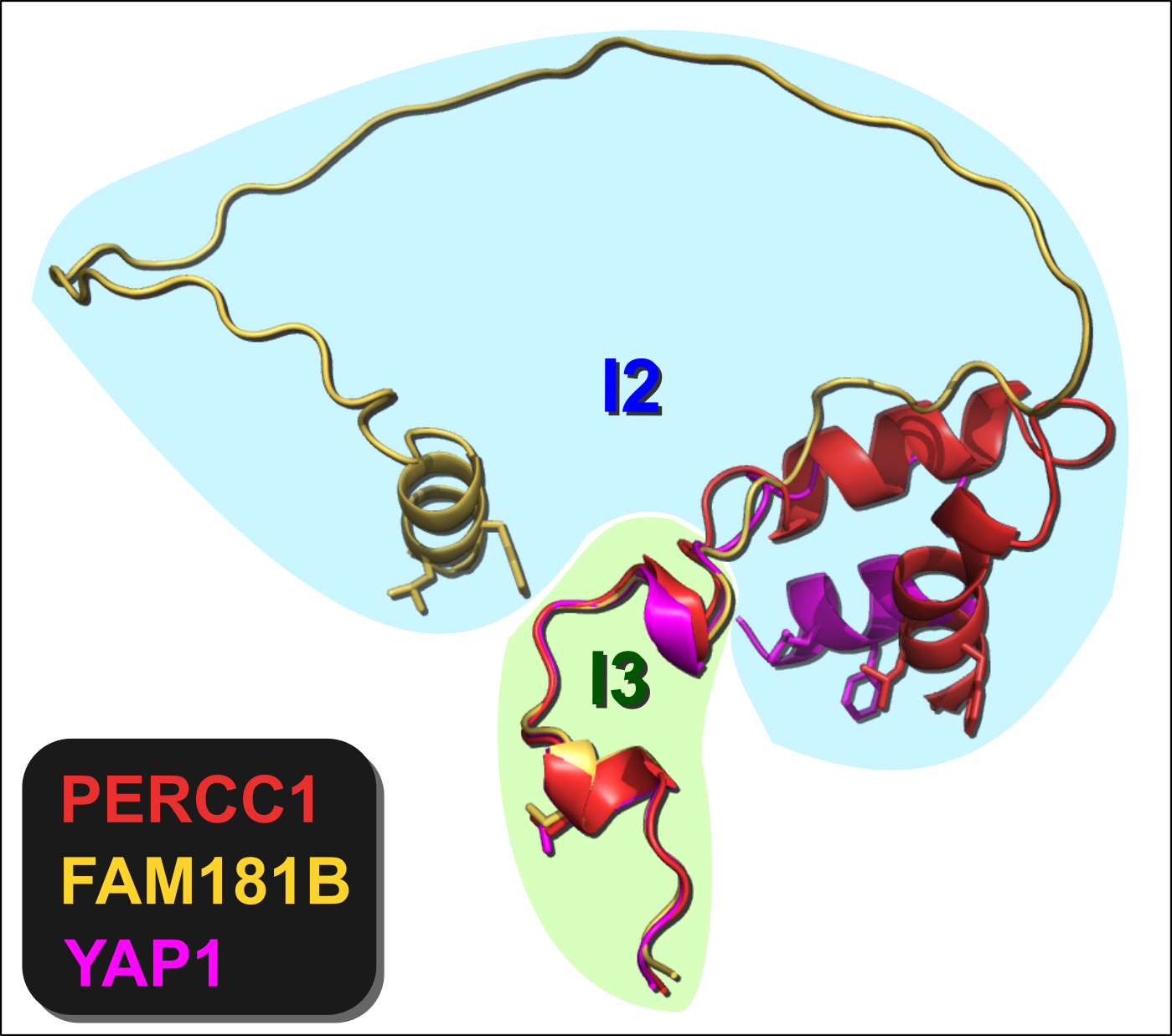

Supplement: vbac008_Supplementary_Data [file vbac008_supplementary_data.zip › FigureS7Percc1.png]

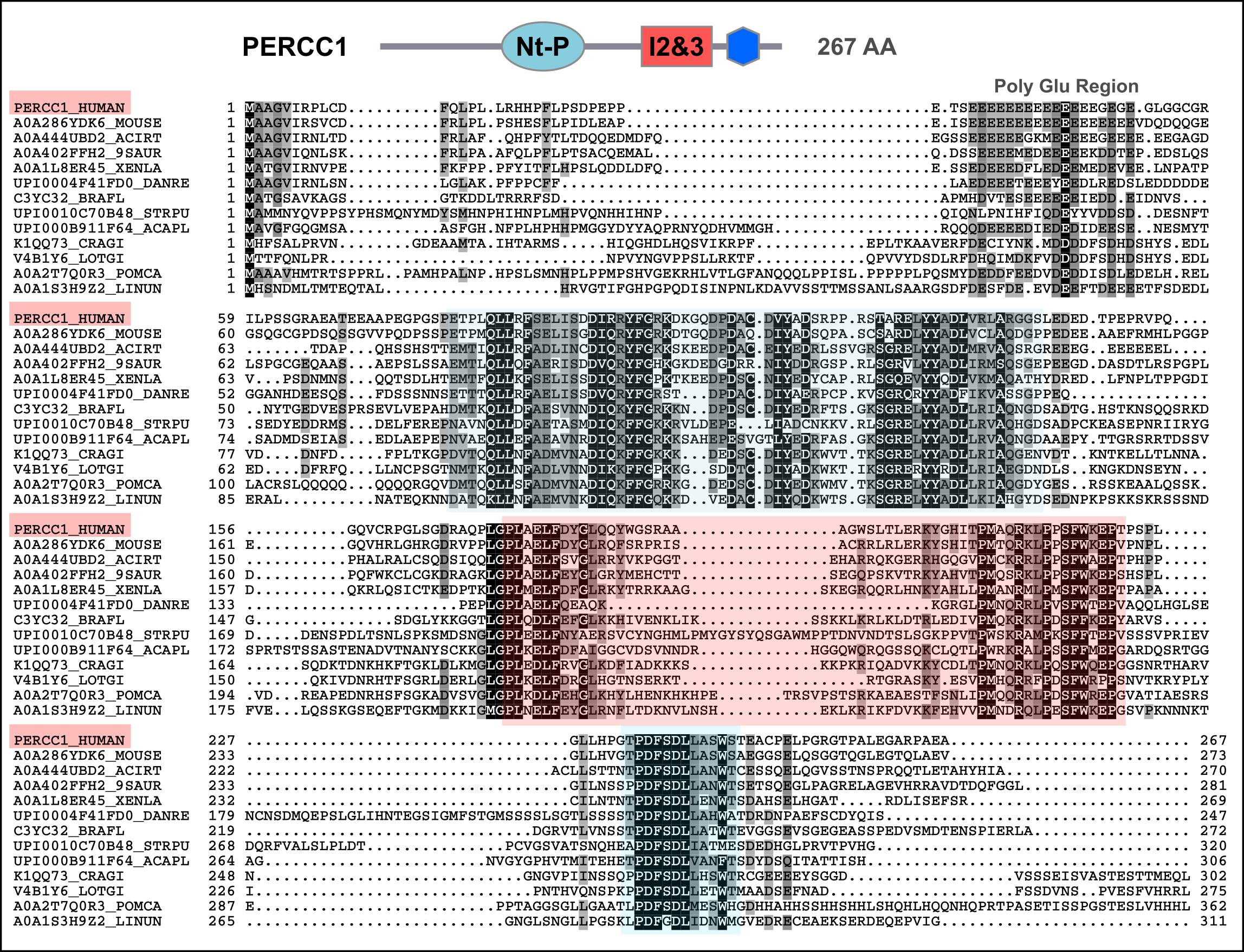

Supplement: vbac008_Supplementary_Data [file vbac008_supplementary_data.zip › FigureS1Percc1.png]
